# Supplementary material for: DNA-based prediction of external ear morphology in the Chinese population: an exploratory study
Source: PeerJ. 2025 Oct 9;13:e20169. doi: 10.7717/peerj.20169 (PMC12515430; doi:10.7717/peerj.20169)

Exploratory DNA-based prediction of external ear morphology in the Chinese population

Supplementary File

Supplementary Tables (Table S1-6, Table S22-23) 2

Supplementary Figures (Fig. S1-5) 6

Table S7-S21 were provided in separate Excel files.

Table S1. Intraclass correlations coefficients (ICCs) of ear trait scores

We calculated the ICC for ear trait scores following the definition of Shrout & Fleiss (1979). Scores from a set of photographs for 50 individuals were used for calculating ICCs for each ear phenotype. The photographs were scored twice by two raters, independently, one week apart. Inter-observer reliability was assessed using ICC(2,k) (Two-Way Random-Effects, Average Measures) based on raters' mean scores, whereas intra-observer reliability was assessed via ICC(3,1) (Two-Way Mixed-Effects, Single Measures) for each rater across two time points.

Trait	Intra-observer 1	Intra-observer 2	Inter-observer
**EP**	0.97	0.83	0.73
**LA**	0.80	0.93	0.76
**LS**	0.76	0.81	0.75
**TS**	0.64	0.72	0.68
**AS**	0.75	0.71	0.63
**ItI**	0.88	0.86	0.77
**SHR**	0.71	0.64	0.59
**PHR**	0.84	0.90	0.74
**FA**	0.55	0.47	0.58
**AHC**	0.60	0.53	0.49
**CHE**	0.72	0.82	0.75
**SCAE**	0.47	0.56	0.52
**DT**	0.92	0.85	0.72

Table S2. Correlation between the ear traits examined

Correlation values are presented in the lower left triangle while corresponding permutation p-values are presented in the upper right triangle. Correlations with significant p-values (<6.41E-4, Bonferroni-adjusted threshold) are highlighted **in bold**.

Traits	EP	LA	LS	TS	AS	ItI	SHR	PHR	FA	AHC	CHE	SCAE	DT
**EP**		6.07E-01	5.85E-08	4.93E-01	9.43E-01	1.05E-04	3.48E-04	1.42E-04	1.40E-04	5.20E-04	3.80E-02	1.64E-01	9.88E-01
**LA**	-0.019		4.75E-01	3.28E-01	1.17E-01	7.02E-01	1.52E-04	5.46E-01	3.80E-01	7.96E-02	3.02E-01	5.36E-01	5.56E-03
**LS**	**-0.207**	-0.028		1.01E-01	3.57E-01	8.00E-06	3.00E-05	1.36E-02	7.51E-01	9.18E-01	1.50E-05	2.10E-01	1.61E-01
**TS**	-0.027	0.038	0.063		3.36E-04	8.79E-04	1.09E-01	2.86E-01	2.00E-01	5.59E-02	2.01E-01	2.44E-02	3.94E-01
**AS**	-0.003	0.060	-0.036	**0.138**		3.40E-05	9.00E-03	9.42E-01	1.00E-02	4.22E-01	3.14E-01	7.67E-01	4.92E-01
**ItI**	**0.149**	-0.015	**-0.171**	-0.128	**-0.159**		7.51E-02	7.93E-02	5.56E-02	4.47E-02	2.83E-02	9.80E-01	3.76E-01
**SHR**	**-0.137**	**0.145**	**0.160**	0.062	0.101	-0.069		1.30E-02	6.67E-01	1.12E-01	5.00E-01	1.80E-01	9.00E-01
**PHR**	**0.146**	-0.023	-0.095	-0.041	0.003	0.068	0.096		5.79E-04	6.16E-04	1.41E-03	6.78E-01	1.23E-03
**FA**	**-0.146**	-0.034	-0.012	-0.049	0.099	-0.074	0.017	**0.132**		2.76E-01	1.14E-01	1.07E-08	9.85E-02
**AHC**	**-0.133**	0.068	0.004	0.074	0.031	-0.077	0.061	**-0.132**	0.042		9.35E-02	2.63E-01	1.01E-01
**CHE**	0.080	0.040	**-0.166**	-0.049	0.039	0.085	0.026	0.123	0.061	-0.065		4.24E-02	5.62E-01
**SCAE**	-0.054	0.024	-0.048	-0.087	0.012	-0.001	0.052	0.016	**0.218**	-0.043	0.078		4.12E-01
**DT**	0.001	-0.107	-0.054	0.033	-0.027	0.034	0.005	0.124	0.064	-0.063	0.022	0.032	

Traits abbreviations

EP: Ear Protrusion

LA: Lobe Attachment

LS: Lobe Size

TS: Tragus Size

AS: Antitragus Size

ItI: Intertragic Incisure

SHR: Superior Helix Rolling

PHR: Posterior Helix Rolling

FA: Antihelix Fold

AHC: Antihelix Curvature

CHE: Crus Helix Expression

SCAE: Superior Crus of Antihelix Expression

DT: Darwin’s Tubercle

Table S3. Correlation between the ear traits and covariates

Sex coded as female=1, male=0. Correlations with significant p-values (<3.85E-03, Bonferroni-adjusted threshold) are highlighted in bold.

	EP	LA	LS	TS	AS	ItI	SHR	PHR	FA	AHC	CHE	SCAE	DT
**Sex**	**-0.209**	**0.127**	**0.219**	0.056	-0.069	-0.081	**0.343**	**-0.178**	-0.034	0.096	-0.041	-0.014	-0.014
**Age**	-0.097	**-0.124**	0.108	-0.057	**-0.137**	-0.016	**-0.136**	0.035	-0.019	**-0.126**	-0.105	0.040	0.010

Table S4. Allele frequencies of SNP markers in the multiplex assays

SNP ID	Major Allele	Minor Allele	Frequency
rs3827760	G	A	0.07
rs17023457	T	C	0.44
rs74030209	C	T	0.35
rs6802174	C	G	0.39
rs10198822	T	C	0.44
rs6699106	C	T	0.32
rs1948400	C	T	0.47
rs17034666	A	G	0.08
rs7812632	C	G	0.15
rs62169501	C	T	0.29
rs3789101	G	C	0.26
rs263156	G	T	0.40
rs1960918	C	T	0.25
rs7771119	C	A	0.34
rs1619249	G	A	0.48

Table S5. Results of Hardy-Weinberg equilibrium analysis

No.	SNP	No of obs. Het	No of Exp. Het	Fisher's p value
1	rs6802174	351	321.2	0.016261
2	rs1960918	273	253.8	0.050359
3	rs62169501	303	278.5	0.022883
4	rs7812632	167	174.9	0.220832
5	rs1619249	411	362.0	1.23E-08
6	rs17023457	430	333.3	6.03E-14
7	rs3789101	293	261.7	0.001862
8	rs10198822	384	332.0	4.59E-05
9	rs3827760	83	90.2	0.042531
10	rs17034666	91	97.1	0.117621
11	rs1948400	455	336.0	2.22E-15
12	rs74030209	320	305.1	0.205964
13	rs263156	429	323.5	6.55E-15
14	rs6699106	320	294.2	0.024378
15	rs7771119	419	301.6	2.44E-15

No of obs. Het: number of observed heterozygote samples

No of Exp. Het: number of expected heterozygote samples

Table S6 Results of linkage disequilibrium analysis

D' values are presented in the upper left triangle while corresponding r^2 values are presented in the lower right triangle.

**SNP**	rs3827	rs1702	rs7403	rs6802	rs1019	rs6699	rs1948	rs1703	rs7812	rs6216	rs3789	rs2631	rs1960	rs7771	rs1619
rs3827		0.059	0.006	0.336	0.037	0.059	0.007	0.458	0.027	0.116	0.012	0.274	0.053	0.259	0.170
rs1702	0.000		0.003	0.106	0.055	0.101	0.238	0.141	0.006	0.016	0.085	0.025	0.143	0.416	0.207
rs7403	0.000	0.000		0.023	0.053	0.037	0.074	0.028	0.067	0.139	0.077	0.042	0.044	0.000	0.072
rs6802	0.014	0.006	0.000		0.071	0.063	0.628	0.145	0.162	0.034	0.006	0.047	0.025	0.197	0.070
rs1019	0.000	0.003	0.001	0.002		0.039	0.072	0.028	0.013	0.864	0.107	0.016	0.193	0.078	0.178
rs6699	0.001	0.006	0.001	0.003	0.001		0.249	0.039	0.147	0.064	0.061	0.109	0.068	0.003	0.015
rs1948	0.000	0.041	0.003	0.281	0.003	0.025		0.113	0.108	0.047	0.050	0.240	0.038	0.319	0.095
rs1703	0.201	0.001	0.000	0.003	0.000	0.000	0.001		0.004	0.037	0.133	0.029	0.010	0.068	0.030
rs7812	0.000	0.000	0.002	0.008	0.000	0.009	0.002	0.000		0.022	0.009	0.058	0.090	0.180	0.082
rs6216	0.003	0.000	0.004	0.000	0.394	0.001	0.001	0.000	0.000		0.145	0.094	0.004	0.033	0.146
rs3789	0.000	0.003	0.004	0.000	0.003	0.001	0.001	0.001	0.000	0.003		0.271	0.028	0.308	0.112
rs2631	0.004	0.000	0.001	0.001	0.000	0.008	0.043	0.000	0.000	0.002	0.017		0.027	0.821	0.278
rs1960	0.001	0.006	0.000	0.000	0.017	0.001	0.001	0.000	0.005	0.000	0.000	0.000		0.165	0.071
rs7771	0.003	0.072	0.000	0.012	0.004	0.000	0.044	0.001	0.003	0.000	0.017	0.517	0.005		0.429
rs1619	0.002	0.034	0.003	0.003	0.025	0.000	0.008	0.000	0.001	0.009	0.004	0.048	0.002	0.087	

SNP abbreviations

rs3827: rs3827760

rs1702: rs17023457

rs7403: rs74030209

rs6802: rs6802174

rs1019: rs10198822

rs6699: rs6699106

rs1948: rs1948400

rs1703: rs17034666

rs7812: rs7812632

rs6216: rs62169501

rs3789: rs3789101

rs2631: rs263156

rs1960: rs1960918

rs7771: rs7771119

rs1619: rs1619249

Table S22. Prediction accuracy parameters obtained for the binomial models including gene interactions with different probability thresholds applied.

**Absent Tragus**		The highest probability approach	p>0.5	p>0.55	p>0.6	p>0.65	p>0.7	p>0.75	p>0.8	p>0.85
AdaBoost	Sensitivity	0.64	0.64	0.77	0.89	0.94	0.95	0.96	1.00	1.00
	Specificity	0.65	0.65	0.71	0.90	0.89	1.00	1.00	1.00	1.00
	PPV	0.71	0.71	0.87	0.95	0.94	1.00	1.00	1.00	1.00
	NPV	0.59	0.59	0.48	0.69	0.77	0.78	0.88	1.00	1.00
	Prediction error*	0.35	0.35	0.21	0.12	0.10	0.05	0.03	0.00	0.00
	Inconclusive	0.00	0.00	0.62	0.88	0.93	0.97	0.97	0.98	0.99
BLR	Sensitivity	0.71	0.71	0.71	0.75	0.75	0.80	0.84	0.80	0.90
	Specificity	0.63	0.63	0.65	0.52	0.61	0.81	0.95	0.96	0.97
	PPV	0.71	0.71	0.74	0.72	0.71	0.85	0.95	0.95	0.96
	NPV	0.65	0.65	0.66	0.52	0.55	0.50	0.47	0.49	0.67
	Prediction error*	0.33	0.33	0.31	0.31	0.31	0.23	0.16	0.22	0.12
	Inconclusive	0.00	0.00	0.19	0.36	0.47	0.66	0.79	0.86	0.93

Table S23. Explanation of genetic terminology.

Abbreviation	Complete Name	Explanation
DNA	Deoxyribonucleic Acid	A double-stranded biological macromolecule that stores genetic information and directs the synthesis of RNA and proteins.
SNP	Single Nucleotide Polymorphism	A genetic variation occurring at a single nucleotide position in the genome, commonly used in genetic association studies.
GWASs	Genome-Wide Association Studies	A hypothesis-free approach to scan the entire genome for genetic variants associated with specific diseases or traits.
MAF	Minor Allele Frequency	The frequency of the less common allele at a specific genetic locus within a given population.
LD	Linkage Disequilibrium	The non-random association of alleles at different loci on the same chromosome, often due to genetic linkage or population history.
PCR	Polymerase Chain Reaction	A molecular biology technique used to amplify specific DNA fragments, enabling the detection of trace amounts of DNA.
SBE	Single Base Extension	A genotyping method that extends a single nucleotide at the 3' end of a primer to identify SNPs.
HW equilibrium	Hardy-Weinberg Equilibrium	A principle stating that allele and genotype frequencies in a large, randomly mating population remain constant from generation to generation in the absence of evolutionary forces.

Fig. S1 Distributions of ear phenotype scores

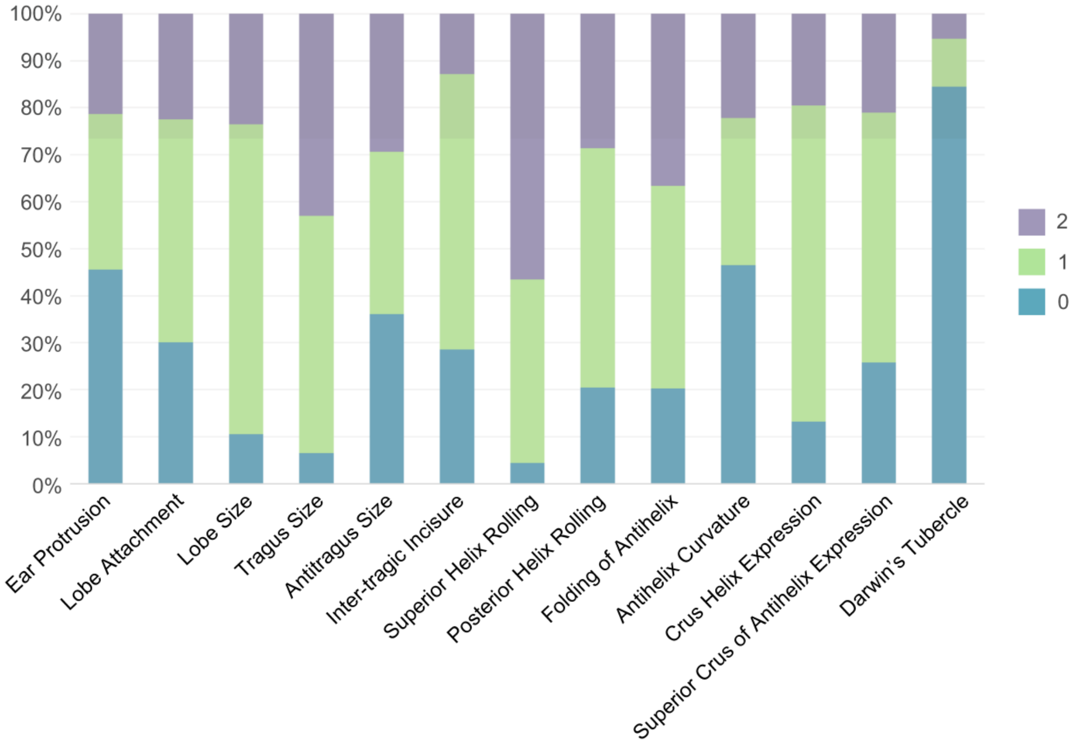

Fig. S2 Overall framework of DNA-based prediction for ear morphology

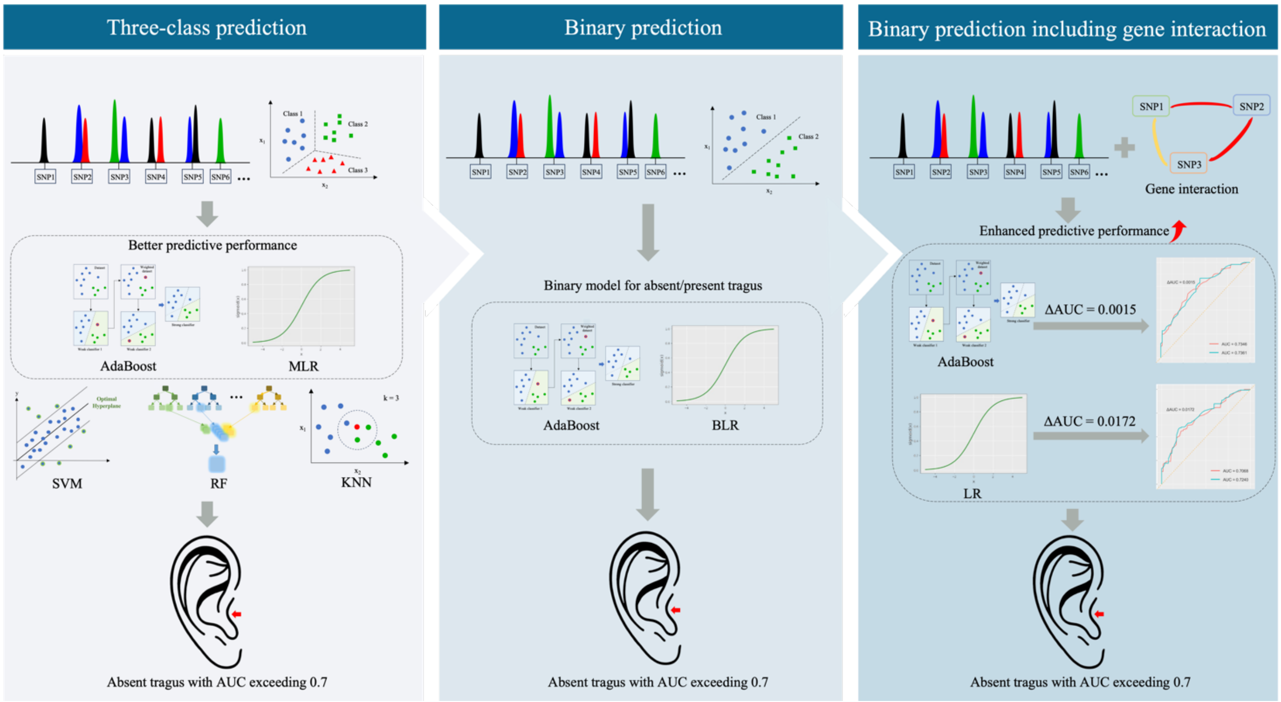

Fig. S3 Entropy-based interaction dendrograms for absent tragus (level_0)

Interaction dendrograms are designed to visualize the interplay between variables. They are constructed using hierarchical clustering, which groups variables with strong interactions near the leaf nodes of the tree structure, thereby intuitively displaying the correlation patterns among these variables. In interaction dendrograms, synergistic effects are typically denoted by red or orange lines, which indicate that when two variables act together, the amount of information they provide increases. Conversely, redundant effects are usually represented by blue or green lines, which suggest that when two variables work in conjunction, the information they offer actually diminishes, potentially because the variables provide similar information.

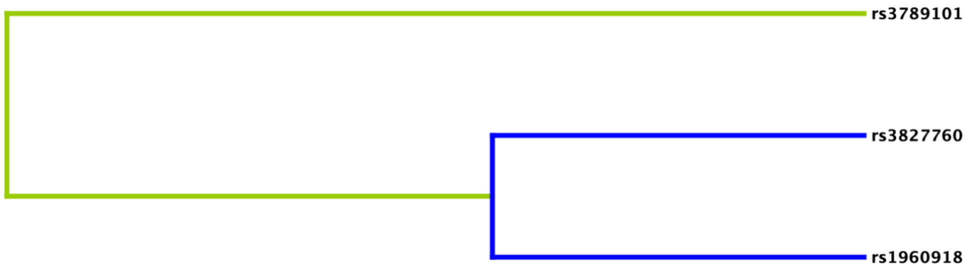

Fig. S4 Average confusion matrices for absent tragus (level_0).

a) Confusion matrices of the AdaBoost model; b) Confusion matrices of the binary logistic regression model.

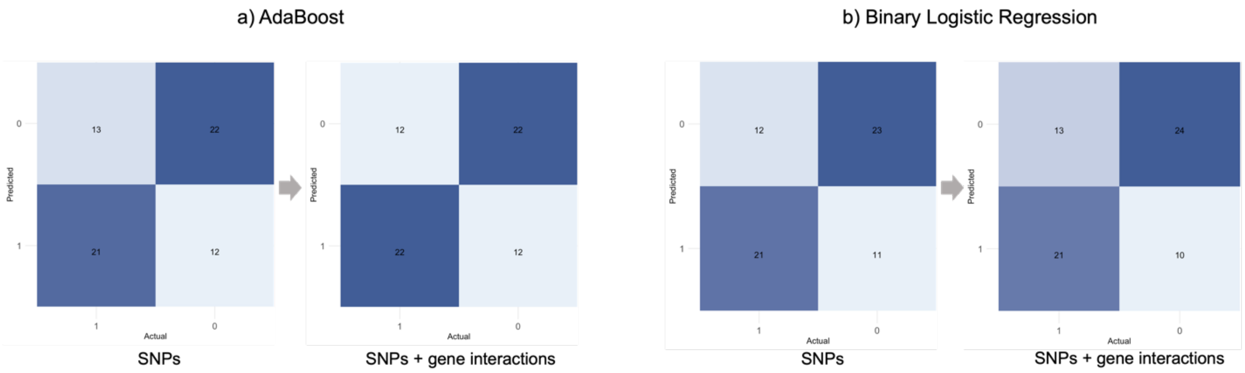

Fig. S5 Average precision-recall curves for absent tragus (level_0).

a) Precision-recall curves of the AdaBoost model; b) Precision-recall curves of the binary logistic regression model. AP: Area under the precision-recall curve.

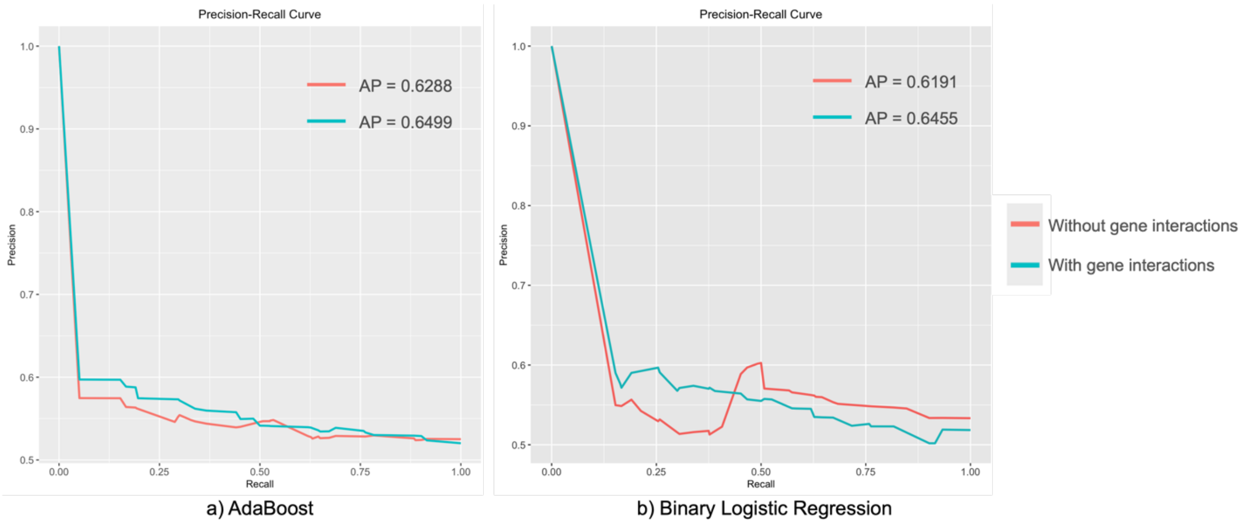

Supplement: Supplemental Information 1 [file peerj-13-20169-s001.pdf]
